# Upgrade of an old drug: Auranofin in innovative cancer therapies to overcome drug resistance and to increase drug effectiveness

**DOI:** 10.1002/med.21872

**Published:** 2021-12-01

**Authors:** Tania Gamberi, Giovanni Chiappetta, Tania Fiaschi, Alessandra Modesti, Flavia Sorbi, Francesca Magherini

**Affiliations:** ^1^ Department of Experimental and Clinical Biomedical Sciences University of Florence Florence Italy; ^2^ Biological Mass Spectrometry and Proteomics Group Plasticité du Cerveau UMR 8249 CNRS Paris ESPCI Paris‐PSL France

**Keywords:** Auranofin, cancer, drug resistance, NF‐kB inhibition, proteasome inhibition, thioredoxin reductase inhibition

## Abstract

Auranofin is an oral gold(I) compound, initially developed for the treatment of rheumatoid arthritis. Currently, Auranofin is under investigation for oncological application within a drug repurposing plan due to the relevant antineoplastic activity observed both in vitro and in vivo tumor models. In this review, we analysed studies in which Auranofin was used as a single drug or in combination with other molecules to enhance their anticancer activity or to overcome chemoresistance. The analysis of different targets/pathways affected by this drug in different cancer types has allowed us to highlight several interesting targets and effects of Auranofin besides the already well‐known inhibition of thioredoxin reductase. Among these targets, inhibitory‐κB kinase, deubiquitinates, protein kinase C iota have been frequently suggested. To rationalize the effects of Auranofin by a system biology‐like approach, we exploited transcriptomic data obtained from a wide range of cell models, extrapolating the data deposited in the Connectivity Maps website and we attempted to provide a general conclusion and discussed the major points that need further investigation.

**Abbreviations**
17AAG: 17‐Allylamino‐17‐demethoxygeldanamycin
2‐DG: 2‐deoxy‐d‐glucose
5‐FU: 5‐fluorouracil
AAL: acute lymphoblastic leukemia
ADE: adenanthin
AF: auranofin
AKT: serine/threonine kinase 1
ANXA5: annexin A5
APL: acute Promyelocytic Leukemia
ATRA: all‐trans‐retinoic acid
BAX: Bcl‐2 associated X‐protein
BCP‐ALL: B‐cell acute lymphoblastic leukemia
BRCA: BReast CAncer gene
BSO: buthionine sulfoximine
CCL: chronic lymphocytic leukemia
CE: celecoxib
cHL: classical Hodgkin lymphoma
CMAP: connectivity Maps
CML: chronic myelogenous leukemia
DLBCL: diffuse large B‐cell lymphoma
DR4/5: death receptor 4/5
DTX: docetaxel
DUBs: deubiquitinases
EGFR: epidermal growth factor receptor
ENZ: enzalutamide
ERK: extracellular signal‐regulated kinases
FDA: Food and Drug Administration
FOXO3: Forkhead Box O3
GCLC: Glutamate‐Cysteine Ligase Catalytic Subunit
GSH: glutathione
GSR: glutathione‐disulfide reductase
Her2: receptor tyrosine‐protein kinase erbB‐2
HER‐2: human epidermal growth factor receptor 2
HNSCC: neck squamous cell carcinoma
HO‐1: heme oxygenase‐1
IAP: inhibitor of apoptosis proteins
IkB: Inhibitor of NF‐κB
IKK: IkB kinase
IL‐6: interleukin 6
IM: Imatinib mesylate
JAK2: tyrosine‐protein kinase Janus Kinase 2
JNK: c‐Jun N‐terminal kinase
l‐ASC: l‐ascorbate
MAPK: mitogen‐activated protein kinase
MCL: mantle cell lymphoma
Mcl‐1: myeloid‐cell leukemia 1
MEK: MAPK/ERK kinase
MLL: mixed lineage leukemia
MM: multiple myeloma
mTOR: mammalian target of rapamycin
MUC4: mucin 4
NAC: N‐acetyl‐l‐cysteine
NF‐κB: nuclear factor kappa‐light‐chain‐enhancer of activated B cells
NONO: non‐POU domain‐containing octamer‐binding
Nrf2: nuclear factor erythroid 2–related factor 2
NSCLC: non‐small cell lung cancer
OC: ovarian cancer
PAI‐2: plasminogen activator inhibitor
PAK1: p21 activate kinase
PB1: Phox and Bem 1
PD‐L1: programmed death‐ligand 1
PI3K: phosphatidylinositol‐3‐kinase
PKCiota: protein kinase C iota
PL: piperlongumine
PRDXI: peroxiredoxin I
Prx3: peroxiredoxin3
PTEN: phosphatase and tensin homolog protein
PTGR1: prostaglandin reductase 1
ROS: reactive oxygen species
SCLC: small cell lung carcinoma
SeC: selenocysteine
Smac: second mitochondria‐derived activator of caspases
SP: side‐population
STAT3: signal transducer and activator of transcription 3
TAK1: transforming growth factor β‐activated kinase 1
TIC: tumor‐initiating cell
TNBC: triple negative tumors
TP53: mutated or
Trx: thioredoxin
TrXR: thioredoxin reductase
TUSC2: tumor suppressor candidate 2
VDAC: voltage‐dependent anion‐selective channel protein
XCT: cystine/glutamate transporter
YAP1: yes‐associated protein 1
ZnPP IX: zinc protoporphyrin IX
γ‐GCL: γ‐glutamylcysteine ligase

## 1 INTRODUCTION

**Figure 1:**
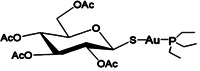
Chemical structure of Auranofin

Auranofin (AF) is a linear gold(I) complex bearing triethylphosphine and thioglucose tetraacetate as ligands (Figure 1). AF has been adopted in clinical use since 1985 for the treatment of rheumatoid arthritis. It is an orally administered drug and is considered safe for human use due to the well‐known toxicity profile. After oral administration, 15%–25% of the drug can be detected in plasma and the serum albumin has been found to carry about 80%–95% of gold circulating in the blood.^1^, ^2^ The pharmacokinetics of AF has been extensively described by Chaffman et al.^3^ The mean pick of AF concentration, following a single 6 mg dose, was attained after 102–120 min and it ranged between of 0.066 and 0.23 µg/ml (corresponding to a molar concentration of 0.1–0.34 µM). Steady‐state plasma gold concentrations following at least 12 weeks of 2–9 mg daily dose of AF were 0.20–1.0 µg/ml, respectively corresponding to 0.3–1.5 µM. More recently in a Phase I human study, where AF was used as an antiparasitic agent at the dose of 7 mg daily (dose recommended in rheumatoid arthritis therapy), gold level in plasma of treated subjects for over a week reached 1–1.5 µM.^4^ The triethyl‐phosphine ligand is lipophilic, conferring membrane solubility to the complex. Once the compound has entered into the cells, the thiol ligand reacts with thiol and selenol groups, with which it forms stable and irreversible adducts.^5^ In mammals most of the effects caused by this drug are thought to be related to its inhibitory activity against the thioredoxin reductase enzymes: TrxR1 and TrxR2.^6^, ^7^, ^8^, ^9^, ^10^ The thioredoxin reductase enzymes play an important role in multiple intracellular processes including DNA synthesis, transcriptional regulation, cell growth and resistance to oxidative agents that induce oxidative stress and apoptosis. An increase of the thioredoxin reductase/thioredoxin (TrxR/Trx) system has been reported in many tumors compared to normal tissues and the ability of certain cancer cells to maintain a highly reduced intracellular environment is correlated with tumor aggressiveness and drug resistance. Furthermore, high expression level of TrxR/Trx system directly correlates with poor prognosis in a variety of cancers including lung cancer^11^ and breast cancer.^12^ Indeed, this system plays a pivotal role in maintaining the reduced and active state of several proteins including phosphatase and tensin homolog (PTEN) that negatively regulates phosphatidylinositol‐3‐kinase/AKT Serine/Threonine Kinase 1 (PI3K/Akt) pathway which is positively involved cell survival.^13^ Several elegant reviews have already discussed the role of TrxR/Trx system under physiological and pathological conditions.^14^, ^15^ Other areas of research for the antiproliferative activity of AF focus on the inhibition of signal transducer and activator of transcription 3 (STAT3),^16^, ^17^, ^18^ nuclear factor‐kappa B (NF‐κB)^18^, ^19^ and protein kinase C iota (PKCiota)^20^, ^21^ signaling. Furthermore, proteasome inhibition and activation of forkhead box O3 (FOXO3) were also reported as possible mechanisms involved in AF cytotoxicity.^22^ Based on this evidence, AF is today object of important drug repurposing plans in anticancer therapy with encouraging results, as summarized in this review. In this context, results obtained by research studies have already been translated into clinical trials to develop new alternative therapeutic strategies. Indeed, despite enormous advances in cancer research and treatment, there are still some types of cancer for which there is no specific therapy and hence, chemotherapy and radiation therapy remain pillars of cancer treatment. Furthermore, in these cases patients often respond only partially to therapy and develop resistance over long‐term treatment. Hence, the development of combination therapies may be useful to eradicate cancer cells that are unresponsive to monotherapy. Up to now, Phase I and II studies on chronic lymphocytic leukemia (NCT01419691) and a pilot study on ovarian cancer (NCT01747798) have been completed, while other clinical trials with AF as monotherapy or in combination with other drugs are ongoing on glioblastoma (NCT02770378), on lung cancer (NCT01737502), on ovarian cancer (NCT03456700). AF is also under investigation for use in infective diseases (NCT02736968 for Giardia protozoa, NCT02961829 for HIV, and NCT02968927 for tuberculosis).

In this review we have reviewed articles that use AF (alone or in combination with other molecules) as an anticancer drug. We focused first on the effect of AF on different types of cancer cells and/or animal models and then we analysed and integrated the results by a system biology‐like approach, with the data available on Connectivity Map website (https://clue.io/cmap). Finally, we attempted to extrapolate general conclusion and discussed the major points that need further investigation. In Table 1 we reported AF effect and mechanism of action on different cancer models (cell lines and/or animal model). Most of the articles are commented on throughout the text.

**Table 1:** Summary of the studies focusing on AF repurposing as single drug or in combination

AF as single drug or in combination	AF concentration and time of treatment^a^	Cell lines	In vivo experiment	Mechanisms/pathways involved	References^b^
*Multiple myeloma*					
AF + NF‐kB inhibitor SN‐50	0.05–0.1 µM AF for 6, 12, 24 h	U266, RPMI8226, IM‐9 cell lines and primary cell lines		Inhibition of IL‐6 induced‐JAK/STAT pathway and NF‐kB activity	^18^
AF + PX‐12 (inhibitor of Trx1)	0–8 µM AF for 24 h (EC50 = 0.05 µM at 24)	RPMI8226, U266 ‐resistant to bortezomib		Oxidative stress (Trx system)	^31^
AF	0–8 µM AF for 24 h	RPMI8226, U266 ‐resistant to bortezomib		Oxidative stress (Trx system) and NF‐kB signaling pathway (NF‐kb p65)	^32^
AF + HO‐1 inhibitor Zinc Protoporphyrin IX	0–4 µM AF for 24 h (EC50 (24 h) = 0.48 µM for RPMI8226, 0.46 µM for U266, and 0.457 µM for OPM2 cells)	RPMI8226, U266, OPM2 and PBMCs		HO‐1 expression through the Nrf2 signaling	^35^
AF + Prima‐1 Met (APR‐246)	HMCLs = 0.25–2 µM AF for 2 days; (EC50 = 0.4 µM). Primary cells = 50 or 500 nM AF for 24 h.	7 HMCLs with a wild‐type TP53 status (MDN, NCI‐H929, NAN9, NAN11, XG3, XG6, XG7), 8 HMCLs with a missense TP53 mutation (JIM3, KMS12PE, LP1, NAN10, OPM2, U266, XG2, XG5) and 3 HMCLs with a TP53 indel leading to the lack of mRNA and/or protein expression (JJN3, L363, NAN7). Primary cell lines		Oxidative stress (ROS/GSH pathway)	^40^
AF + ([Au(d2pype)2]Cl)	0.25‐4 µM AF for 24 h Mice = AF 5 mg/kg, (5 times a week, for 2 weeks).	RPMI8226, U266 and JJN3 myeloma cells	human RPMI8226 xenograft model	Oxidative stress (Trx system)	^42^
AF + seventy‐six FDA‐approved oncology drugs and emerging therapeutics	MM cell lines = 0.01–10 µM AF for 24 and 72 h. Primary cells = 0.01–10 µM AF for 24 h (EC50 below 100 nM).	25 multiple myeloma (MM) and 15 non‐Hodgkin's lymphoma cell lines and in 113 primary MM samples		Drug direct screening	^43^
*Leukemia*					
AF	AF 3–7.5 mg/kg, every fourth day, daily, and twice daily		C57BL x DBA/2 F, mice inoculated with the lymphocytic leukemia P388		^44^
AF	AF 1.5–96 mg/kg/daily for days 1–5. Optimal activity with 12 mg/kg/daily for days 1–5.		C57BL x DBA/2 F, mice inoculated with the lymphocytic leukemia P388		^45^
AF + all‐trans retinoic acid (ATRA)	0.1–1 µM AF for 4 days or 24 h. 0.3 µM AF and 5 nM ATRA	NB4 cell		Oxidative stress (Trx system)	^48^
AF	0.5‐2 µM AF for 1–12 h	HL‐60 cell		p38 MAPK activation	^52^
AF + all‐trans retinoic acid (ATRA) and 1,25(OH)2 vit D3	NB4 cells and APL blasts = 0.3–0.5 µM AF for 4–5 days. HL‐60 cells = 0.5 µM AF and 3 nM 1,25(OH)2 vit D3 in combination for 3 days.	NB4 cells and HL‐60, APL blasts from leukemia patients		Enhancing histone acetylation	^49^
AF	Cells = 0.125‐4 µM AF for 24 h; 1 µM for 24 and 48 h. Mice = 10 mg/kg (5 days/week for 2 weeks)	Human chronic B‐cell leukemia, MEC‐1 cells, primary CD19 + CLL cells	TCL‐1 transgenic mice genotype (Tcl1‐g:p53 ^‐/‐^)	Oxidative stress (Trx system) and Endoplasmic Reticulum Stress	^53^
AF	Cells = 0.5‐2 µM AF for 6,12,24 and 48 h. Mice = AF 7 mg/kg/day for 12 days	KBM5 (Bcr‐Abl wild‐type) and KBM5‐T315I (Bcr‐Abl‐T315I) Chronic myelogenous leukemia cells	mouse imatinib‐resistant xenograft models	Bcr‐Abl signaling and proteasome‐dependent caspase activation	^57^
AF + [Au(d2pype)2]Cl	0.25–4 µM AF for 24 and 48 h	K562 and KU812 CML cell lines sensitive and resistant to imatinib		Bcr‐Abl signaling and Trx system	^60^
AF + Erastin and BSO	0.1–2 µM AF for 24 h	Human T‐ALL (Jurkat, Molt‐4) and precursor (pre)‐B‐ALL		Oxidative stress pathways	^63^
AF + adenanthin	BCP‐ALL cell lines = 0.0625–2 µM AF for 48 h. Primary leukemic BCP‐ALL blasts = 0.0156–2 µM AF for 92 h. Normal PBMC = 0.0125–2 µM AF for 4 days. Mice = AF 10 mg/kg, once daily for 3 weeks	Human BCP‐ALL cell lines (697, REH; SEM; SD1; BV17;, SUP‐B15) and B‐cell lymphoma cell line RL, primary BCP‐ALL blasts or their primografts	patient‐derived xenograft model	Oxidative stress pathways	^65^
AF + sorafenib	EC50 = 0.65 µM in Cells = MV11 and 0.71 µM in MV‐11R (sorafenib resistant) Mice = 10 mg/ml AF, 10 mg/ml sorafenib	MV‐11, MV‐11R	MV‐11R and MV‐11 xenograft	TrxR3 inhibition	^66^
*Lymphoma*					
AF	0.5–4 µM AF for 30–40 min and 6, 8, 24 h	Jurkat T‐lymphoma and U937 monocytic cell lines			^67^
AF + doxorubicin, cisplatin, and gemcitabine	Cells = 0.1‐10 µM AF for 24 or 48 h. Mice = 10 mg/kg, 3 times a week	L‐1236, L‐428, KM‐H2, HDLM‐2, and L‐540 cHL‐derived cell lines; L‐540 gemcitabine‐resistant and HDLM‐2 brentuximab resistant cells	L‐540 gemcitabine resistant–derived tumor xenografts,	Oxidative stress pathways	^68^
AF + BSO	0.1–3 µM AF for 24 h. 100 nM AF and 5 µM BSO in combination for 18 h	human DLBCL cell lines, SUD‐HL6 and OCI‐LY10, the MCL cell lines, Rec‐1 and Granta, and the MM cell lines, U266 and KMS‐12‐PE, primary MCL‐derived cells		TrxR and NF‐κB signaling	^69^
AF + l‐ascorbate (l‐ASC)	Cells = 0.125–0.6 µM AF for 48 h. 0.5 µM AF and 200µM l‐ASC in combination for 1–6 h. Mice= AF (2.5 mg/kg once daily) and l‐ASC (3 g/kg twice daily) from day 2 to 7 and day 10 to 15 after cell inoculation	primary chronic lymphocytic leukemia B‐cells	murine B‐cell lymphoma model		^70^
AF	Cells = 0.075‐5 µM for 72 h (EC50 assay). PDX model = 50 mg/kg, daily for 21 consecutive days after 3 days of tumor engraftment	OCI‐Ly8, OCI‐Ly7, and Su‐DHL‐10; OCL‐Ly3, OCI‐Ly10, U2932, TMD8, and HBL‐1); Z‐138, JVM‐2, Mino, Maver‐1, Jeko‐1, and Jeko‐R	TP53‐mutated DLBCL PDX model	TrxR/Trx system	^71^
*Breast cancer*					
AF + BSO + 2DG and 17AAG	0.5 µM AF, 0.5 µM 17AAG, 20 mM 2DG, 1 mM BSO for 24 h	MDA‐MB 231 and SUM159		Oxidative stress (GSH and Trx oxidation)	^72^
AF + 2DG + DHEA	1 µM AF, 24 h; 20 mM 2DG, 24–48 h; DHEA, 300 µM, 24–48 h	MDA‐MB23		Oxidative stress	^73^
AF	1 µM AF, 12 h (for STAT3 inhibition)	MDA‐MB 231		Inhibition of STAT3 phosphorylation through ROS‐dependent mechanism	^17^
AF + Vitamin C	Cells =6 µM AF, 1 h Mice = AF 10 mg/kg+ Vitamin C 4 g/kg; AF 5 mg/kg + VitaminC 4 g/kg or AF	MDA‐MB‐231	MDA‐MB‐231 xenograft	Oxidative stress	^74^
AF + Vitamin C + menadione	05–1 µM AF for 24 h 3, 24 μM Menadione 50, 100 μM l‐Acs	MDA‐MB‐231, HCC1806		Oxidative stress, inhibition of PRDXI activity	^75^
AF	Cells = EC50 values of 1.5 μM (24 h) and 0.41 μM (48 h) Mice = 6 mg/kg/day for 21 days	MCF‐7	MCF7 xenograft	Inhibition of DUBs	^76^
AF + anti PD‐L1 antibodies	EC50 = 0.5–2 µM depending on TNBC cell lines Mice = 5 mg/kg, 14 days	SUM159 and MDA‐MB‐231	4T1.2 model, MDA‐MB‐231 xenograft and patient‐derived tumor xenograft	Oxidative stress increased, increased EGFR and ERK1/2 phosphorylation, decreased STAT3 phosphorylation	^77^
AF + BSO and radiation	Cells = 0.25–0.5 μM AF for 1–3 h, 0.1 mM BSOfor 24 h Mice = BSO 450 mg/kg and AF 1.7 mg/kg 2 h, before irradiation	MDA‐MB 231 and SUM159	MDA‐MB‐231 xenograft	Oxidative stress	^79^
AF	0.1–5 μM AF	MDA‐MB‐231	MDA‐MB‐231 xenograft 4T1.2 model	NONO expression inhibition	^80^
AF + mesupron (Urokinase‐type plasminogen activator inhibitor)	EC50 (24 h) = 0.7 μM on MDA‐MB; 231 and 0.2 μM on MCF‐7; 10 μM mesupron + 0.125 μM AF	MDA‐MB‐231, MCF‐7		Oxidative stress, downregulation of Akt phosphorylation	^145^
AF + Nutlin‐3a	0.5 μM AF, 2.5 μM Nutlin‐3a	MCF4, MDA‐MB 231		Oxidative stress	^146^
AF + R428 (Axl inhibitor)	EC50 (48 h) = 0.6 μM on MDA‐MB‐231 and 0.47 μM on MCF‐7 2.5 μM R428 + 0.5 μM AF	MDA‐MB‐231, MCF‐7 cells		Oxidative stress	^147^
*Colon and gastric cancer*					
AF + 5Z‐7‐oxozeaenol	Cells = 500 nM AF pretreatment, then 5 µM 5Z‐7‐oxozeaenol. Mice = 1.6 mg/kg AF and 15 mg/kg 5Z‐7‐oxozeaenol for 5 consecutive days, followed by 2 days of AF only, and then 4 more consecutive days of combination treatment.	HCT116 and SW620	SW620 xenografts	Oxidative stress (Trx and GSH systems)	^82^
AF + celecoxib	Cells =1 µM AF; 1 µM AF + 10 µM celecoxib in combination for 24, 48 h. Mice = AF 10 mg/kg; CE 20 mg/kg; CE 60 mg/kg; AF 10 mg/kg + CE 20 mg/kg; AF 10 mg/kg + CE 60 mg/kg	DLD‐1, HCT116, and HT‐29	DLD‐1 xenografts	Oxidative stress (Trx system)	^83^
AF + 5‐FU	Cells = 5–10 μM 5‐FU + 0.4 μM AF, 24 h Mice = 50 mg/kg/5‐FU once every 4 days, +6 mg/kg/d AF	5‐FU‐resistant SW620 and HCT‐8	SW620/5‐FU xenografts	FoxO3‐activation and TR1‐inhibition	^84^
AF	Organoids = 2 µM AF. Mice = 10 mg/kg three times a week for 3 weeks	CT26 and IEC6	Human colon organoid, CTC26 xenografts	RE stress, UPR response mediated apoptosis	^86^
AF + piperlongumine (PL)	EC50 (24 h) = 2.3 μM AF in BC‐823, 1.8 μM in SGC‐7901 and 2.7 μM KATO III Mice = 2 mg/Kg/day AF + 4 mg/Kg/day PL	BGC‐823, SGC‐7901 and KATO III	SGC‐7901 xenografts	ROS mediated RE stress	^87^
AF	0.5 μM AF, 72 h	SW480 and HCT116		PKC iota inhibition	^148^
*Lung cancer*					
AF + BSO + carboplatin	Cells = 0.5 μM AF for H292, 5 μM AF for A459 100 μM BSO, 20 mM 2DG, 2 μM carboplatin for clonogenic survival Mice = 15 mg/kg carboplatin, 450 mg/Kg BSO, 1.6 mg/g AF for 6 days	A549, H292	A549 tumor xenografts	Oxidative stress (Trx system)	^89^
AF + SeC	Cells = 6 µM AF, 6 h after SeC 8 µM, 24 h Mice = 5 mg/kg SeC+2 mg/kg AF, 8 doses.	A549	A549 tumor xenograft	Down regulation of ERK and Akt phosphorylation, p38 and JNK phosphorylation doesn't affected	^90^
AF + MK2206 (Akt inhibitor)	Cells = 0.1 µM AF, 1‐3 µM MK2206 Mice = 25 mg/kg+ MK2206 5 mg/kg AF	HCC193 and HI993	H1993 tumor xenografts nude mice	ROS induction and JNK activation	^92^
AF	Cells = 0.5 µM, 12‐24 h Mice = 10 mg/kg/day	HCC366, Calu3	Calu3 tumor xenograft	PI3K/Akt/mTOR pathway and NRF2‐mediated oxidative stress response inhibition	^93^
AF and TUSC2 gene delivered by nanovesicles + erlotinib	Cells = 0.5–0.6 μM AF, 1 μM erlotinib, 72 h Mice = 10 mg/kg AF five times per week, for two weeks, 30 mg/kg Erlotinib orally feed daily with a total of 8 times	Calu‐3, Calu‐6 and H522	EGFR TUSC2‐deficient human H1299 cells xenograft	NRF2‐mediated oxidative stress response	^94^
AF + adryamicin	EC50(24 h) = 4 µM AF Several different combination Mice = AF 10 mg/kg, and ADM 5 mg/kg, once a week for 6 week	A549 and NCI‐H460 (these cell lines contained an elevate percentage of SP cells)	A549 tumor xenograft	Selective inhibition of SP cell growth by ROS dependent mechanism; ATP depletion through direct hexokinase inhibition	^95^
AF	Cells = 0.5 μM AF for 12‐48 h Mice = 10 mg/kg/day	A459 knocked out of GSR	Patient‐derived xenografts	Oxidative stress	^97^
AF + ibrutinib (EGFR inhibitor)	Cells = 0.25 µM AF, 0.1–0.3 µM ibrutinib, 24‐48 h Mice = 5 mg/kg AF + 25 mg/kg ibrutinib	EGFR wild‐type NSCLC cell lines (Calu3 and H460) EGFR mutant lines, sensitive and resistant to ibrutinib	H1975 tumor xenograft	Akt inhibition	^98^
AF + IPA3 (PAK1 inhibitor)	15 µM AF for 6 h IPA3 5 µM Mice = 10 mg/kg AF + 10 mg/kg IPA3	HCC827 (EGFR mutated), H23 (KRAS mutated) and H520 (PAK1 overexpression)	HCC827, H23, or H520 xenograft nude mice	PKCι signaling inhibition	^99^
AF + cisplatin	Cells = 1 µM AF; 1 µM cisplatin, 4 h Mice = 10 mg/kg/day AF + 3 mg/kg/day cisplatin	H69 and H196 (cisplatin resistant)	H69 xenograft nude mice	ROS induced apoptosis	^100^
AF + KU55933 (Ataxia ‐telangiectasicia mutated protein, ATM ibhibitor)	AF 2 µM in A549; 10 µM in MLF + 10 µM ATM, 4 h	A549, MLF		Oxidative stress	^149^
AF	3–4 µM at 24 h	Several different cell lines		Mitochondrial damage and apoptosis	^150^
*Ovarian cancer*					
AF	EC50(24 h) = 1.4 µM (2008), 1.03 µM (C13)	cisplatin sensitive (2008) and its cisplatin‐resistant variant (C13)		Oxidative stress	^102^
AF + selenite	1 µM AF, 0.5 µM selenite for 48 h	cisplatin sensitive (2008) and its cisplatin‐resistant variant (C13)		Redox unbalance	^103^
AF	0.5 µMAF for 24 h	A2780		Variation of amount of proteins involved in protein degradation and redox homeostasis (proteomic study)	^104^
AF	0.5 µM AF for 24 h	A2780 cisplatin‐resistant		Variation of amount of proteins involved in protein degradation and redox homeostasis (proteomic study)	^105^
AF	EC50 (ES2 TICs) = 0.192 µM, 5 days Mice = 12 mg/kg, 6 days	ES2 and Skov3 cultured in stem cell medium and developing tumorigenic TIC (tumor‐initiating cell) phenotype	ovarian cancer orthotopic mouse model	PKCι signaling inhibition	^106^
AF	0.1 µM AF for48 h	SCOV3		Caspase‐3‐mediated apoptosis in FOXO3‐dependent manner	^22^
AF + MUC4 silencing	0.025 µM AF for 72 h	SKOV3		Inhibition of Her2/Akt/FOXO3 pathway	^110^
AF + BRCA1 silencing	2 µM AF for 18 h	SKOV3, OVCAR5		Double strand brakes in ROS dependent manner	^112^
AF + HSP90 inhibitor (AUY92)	1 µM AF, 0.1 µM AUY92, 24 h	A1847, A2780, OVCAR8, OVCAR4, PEO4, SKOV3		DUBs inhibition	^113^
AF	Cells = 0.7 µM, 48 h Mice = 15 mg/kg three times a week for 2 weeks	A2780	A2780 orthotopic and subcutaneous xenograft mice	TrxR inhibition	^114^
*Other cancers*					
Hepatoma					
AF	2 µM, 6 h	HEPG2		Inhibition of IL‐6 mediated activation of JAK1‐STAT3 pathway	^16^
AF + Disulfiram	10 μM DSF, 0.2 μM AF or their combination for 24 h	SMMC‐ 7721 and HepG2	HepG2 or SMMC‐7721 xenografts	proteasome inhibition, induction of ER stress	^115^
AF + Sorafenib	Different concentration depending on cell lines. Mice = 6 mg/kg/d AF for 7 days	Huh7, MHCC97L, Hep3B, HepG2, PLC/PRF/5)	Hydrodynamic tail‐vein (HDTV) injections of CRISPR‐Cas9‐KO p53 and PTEN plasmids in C57BL/6 N mice	TrxR1 inhibition	^117^
AF + morin	1 µM AF,100‐200 µM morin, 24 h	Hep3B		Extrinsic and intrinsic apoptosis pathways	^118^
AF + sulforaphane	0.5, 1, 1.5, and 2 μM AF or 2.5, 5, 7.5, and 10 μM sulforaphane for 24 h	HepG3		PI3K/Akt Signaling Pathway	^119^
AF	EC50 = 0.43 (24 h) and 0.17 μM (48 h) Mice = 6 mg/kg/day for 21 days	HEPG2	HEPG2 xenograft	DUBs inhibition	^76^
*Prostate cancer*					
AF	2.5 µM AF for 24 h	PC‐3		induction of Annexin A5 expression	^121^
AF	1 µM AF for 24 h	PC‐3		Annexin A5 mediated ihibition of COX‐3	^122^
AF	0.5–1 µM for 24 h	PC‐3		Annexin A5 mediated ihibition of PAI‐2	^123^
AF	Cells = 1 µM AF for 24 h Mice = 6 mg/kg/d	LNcap and 22RV1	22RV1 xenograft	Inhibition of DUBs and androgen receptor degradation	^125^
AF + enzalutamide (ENZ) and docetaxel (DTX)	Cells = 2 µM AF for 12 h Mice = 5 mg/kg/d AF 10 mg/kg/d ENZ5 10 mg/kg/d, 5 days per week DTX once a week	cells resistant to DTX and androgen‐deprivation therapy	R1‐DDR (cells resistant to DTX and ENZ) xenograft	decrease the AR3‐E2F1 axis	^126^
AF	EC50 = 2.18 µM and 0.64 µM PNANC‐1 nutrient sufficient and deprived Mice = 12.5 mg/kg/5 times weekly for 5 weeks	PANC‐1	xenograft models of human PSN‐1	TrxR1 inhibition	^127^
AF + BSO + 2DG and 17AAG	0.5 µM AF for 24 h, 17AAG 20 mM 2DG 15 mM, BSO 1 mM for 24	PC‐3		Oxidative stress	^72^
AF	1 µM AF for 24 h, 20 mM 2DG for 24–48 h, 300 µM DHEA for 24–48 h	PC3, DU145		Oxidative stress	^73^
*Head and neck squamous cell carcinoma*					
AF + BSO	Cells = 0.5 µM AF + 1 mM BSO Mice = 400 mg/kg/d BSO + mg/kg/d AF for 10 day	FaDu, Cal‐27 and SCC‐25	Cal‐27 xenografts	Oxidative stress	^128^
AF + BSO	Cells = 0.1–0.5 μM AF + 5–25 μM BSO Mice: 2 mg/Kg/d AF + 450 mg/kg/d BSO	Several cell lines	HN3‐cisplatin resistant xenografts	Oxidative stress	^129^
*Osteosarcome and Ewing sarcome*					
AF	Cells = EC50(48 h) = 0.7 µM for KHOS, 1.3 µM for MG‐63 Mice = 0.1 mg/kg AF + 2.5 mg/kg vorinostat), or + 0.1 mg/kg rapamycin 5 days per week for 3 weeks	KHOS/NP and MG‐63	KHOS/NP xenograft	Apoptosis induction	^130^
AF + ganetespib	Mice = 12 mg/kg through AF once a day for 5 days per week, and 150 mg/kg ganetespib once weekly	Several cell lines of Ewing sarcome	A673 xenografts		^131^
Melanoma					
AF	Cells = 1–4 µM for 2 h 0.0125–0.2 µM for 2 h EC50(2 h) = 6.5 µM in stationary cell population; 7 µM in logarithmic cell population Mice = 2–24 mg/kg/daily on days 1 through 5.	B16 melanoma cell lines	C57BL x DBA/2 F, mice inoculated with B16 melanoma cells		^45^
AF + MJ25 (2‐{[2‐(1,3‐benzothiazol‐2‐ylsulfonyl)ethyl]thio}−1,3‐benzoxazole)	0.1–5 µM AF for 6, 12, 24 h	human melanoma cell line ARN8 stably transfected with RGCΔFosLacZ and its parental cell line A375; HT‐144 melanoma cells and several others cell lines		TrxR inhibition	^132^
AF	0.25–10 µM AF for 48 h (most potent anti‐melanogenic activity =1 µM)	B16F10 and MNT‑1 melanoma cells		Melanogenesis inhibition through by different mechanisms, including reduction of intracellular tyrosinase enzyme activity, reduction of cAMP levels and increase of immature melanosomes.	^133^
*Cervical cancer*					
AF	EC50 = 2 µM AF for 24 h	HeLa		Oxidative stress	^134^
AF + 2‐DG + BSO	Cells = 0.050 µM AF, 10 mM 2‐DG, 500 mM BSO for 24 h Mice = 400 mg/kg 2‐DG, 200 mg/kg BSO, and 1.5 mg/kg AF 3 times per week, over a period of 35 days.	SiHa, Caski, C33A, ME180	SiHa and Caski xenografts	Oxidative stress	^135^

*Note*: The principal mechanisms or pathways proposed as potential targets are reported.

^a^ AF concentrations, as single drug or in combination, used for determination of EC50 (if calculated in the study) or in key experiments, such as apoptosis and cytotoxicity determination, are reported.

^b^ References include also studies not specifically described in the text.

The bibliographic research was limited to studies published from 1980 to 2020 and found in PubMed and Google Scholar databases. Keywords used to retrieve documents were “Auranofin and cancer.” In PubMed the option “Best match” was applied. The word “auranofin” had to be cited in the title, in the abstract or among keywords. Studies performed with auranofin analogues, but not auranofin, were not included.

## 2 AF REPURPOSING IN CANCER THERAPY

### 2.1 Blood cancers

AF has been shown to exert a significant anticancer activity on several different types of blood cancer cells, namely myeloma, leukemia, and lymphoma. Indeed, as reported in the introduction, AF is currently in phase II clinical trials for the treatment of chronic lymphocytic leukemia.

#### 2.1.1 Multiple myeloma (MM)

MM is the second most common hematological malignancy after non‐Hodgkin's lymphoma, and is characterized by the presence of differentiated plasma cells (PCs) in the bone marrow.^23^ Standard therapies for MM are based on a combination of proteasome inhibitors (bortezomib and carfilzomib) and immunomodulatory agents (thalidomide and lenalidomide).^24^, ^25^, ^26^ However, due to acquired drug resistance and the heterogeneity of myeloma cells, MM remains incurable. Therefore, new therapeutic approaches are needed to overcome drug resistance and improve clinical outcomes in the treatment of MM.

The first study dealing with the potential use of AF to treat MM was carried out by Nakaya et al.^18^ They selected this gold(I) compound based on its well‐known anti‐inflammatory and immunosuppressive properties through the inactivation of NF‐κB signaling.^19^, ^27^ It has been reported that chemoresistance in MM is associated with constitutive activation of NF‐κB and tyrosine‐protein kinase Janus Kinase 2 (JAK2)/STAT3 signaling.^28^ Consequently, Nakaya et al. examined whether AF could exert anticancer activity through the inhibition of these signaling pathways. They carried out the analysis on the human myeloma cell line U266 confirming some of the experiments also in the RPMI8226 and IM‐9 cell lines as well as in cells isolated from bone marrow samples of three patients. The results pointed out the ability of 0.05 µM AF, after 24 h of treatment, to arrest cell cycle in subG1 phase followed by apoptosis via both extrinsic and intrinsic pathways. Apoptosis was achieved by the inhibition of interleukin‐6 (IL‐6) induced‐JAK/STAT3 pathway and the downregulation of the antiapoptotic induced myeloid leukemia cell differentiation protein (Mcl‐1), one of the key proteins for survival of myeloma cells.^29^, ^30^ Furthermore, Nakaya et al. demonstrated that the combinatory treatment with 0.05 µM AF and the specific NF‐κB inhibitor SN‐50 potentiates the downregulation of Mcl‐1 and consequently the apoptotic effect. Raninga et al. showed for the first time a positive correlation between myeloma growth, chemoresistance and high Trx1 and TrxR1 expression levels.^31^ They used AF as a selective inhibitor of TrxR1 and the compound PX‐12 to target Trx1. AF (1–4 µM) and PX‐12 treatment disrupted intracellular redox homeostasis in myeloma cells (RPMI8226 and U266) leading to cell growth arrest and activation of the apoptotic pathway. Therefore, they proposed to consider the inhibition of Trx1 and TrxR1 as a potential novel strategy to treat drug‐resistant MM. In a subsequent study Raninga et al. sought to analyze in more depth the role of TrxR1 in the acquisition of bortezomib (first proteasome inhibitor to be approved by the FDA) resistance in MM.^32^ It is known that hypoxia is a key factor in the acquisition of bortezomib resistance even if the molecular mechanisms involved are still to be elucidated.^33^, ^34^ The authors first showed, by using the gene expression data set (GSE4581), that higher TrxR1 levels were associated to acquired drug resistance and to decreased myeloma patient survival. Second, they found an overexpression of TrxR1 in hypoxic myeloma cells RPMI8226 and U266 resistant to bortezomib, suggesting a possible involvement of this enzyme in the hypoxia‐induced bortezomib resistance. Thus, they used AF (1–4 µM) as a selective TrxR1 inhibitor in hypoxic myeloma cells. The results pointed out that TrxR1 inhibition overcame hypoxia‐induced bortezomib resistance. Accordingly, they demonstrated that hypoxia increased NF‐κB subunit p65 nuclear protein levels and that AF‐induced TrxR1 inhibition, decreased both NF‐κB p65 protein and messenger RNA (mRNA) levels as well as mRNA levels of downstream NF‐κB regulated genes, Survivin and Cyclin D1. Based on these data, the authors proposed TrxR1 inhibitors, such as AF, as single drug or in combination therapy to bypass bortezomib resistance and to increase myeloma patient survival. In the same year Raninga and colleagues investigated a possible crosstalk between TrxR and heme oxygenase‐1 (HO‐1) in multiple myeloma cell lines RPMI8226, U266, and OPM2, aiming to demonstrate the need to target multiple antioxidant systems.^35^ HO‐1 participates in hemoglobin degradation and catalyzes the conversion of intracellular heme into biliverdin, free iron, and carbon monoxide.^36^ In turn, biliverdin is reduced by biliverdin reductase to bilirubin which has anti‐inflammatory, antioxidative, and antiapoptosis properties.^37^ HO‐1 expression and activity has been found increased in several types of cancer implying its potential involvement in chemoresistance. Moreover, HO‐1 expression is regulated by many transcription factors including NF‐κB^38^ and nuclear factor erythroid 2‐related factor 2 (Nrf2)^39^ but its regulation in myeloma cells is unclear. The obtained results pointed out that TrxR inhibition by a sublethal concentration of AF (1 µM) induced HO‐1 expression through the Nrf2 signaling pathway without leading to apoptosis. The study demonstrated that this increase was a compensatory effect to the decrease of TrxR activity and protected cells from undergoing apoptosis. Indeed, only a combinatory use of HO‐1 inhibitor Zinc Protoporphyrin IX (ZnPP IX) and a sublethal AF dose triggered apoptosis in myeloma cells. The idea of a combinatory use of AF in chemoresistant MM treatment was evaluated by Tessoulin and colleagues.^40^ The aim of the study was to overcome chemoresistance in p53 or Bax/Bak mutated myeloma cells by targeting oxidative stress. Prima‐1Met (APR‐246) is a compound able to interact with mutated p53 protein restoring its tumor‐suppressor function and triggering cell death in various cancer types. Since Prima‐1Met has been shown to target TrxR1,^41^ the authors investigated whether AF could display the same anticancer activity on a panel of 18 myeloma cell lines and in 10 primary cells from patients with multiple myeloma or plasma cell leukemia. The results confirmed the ability of both AF (0.4 µM) and Prima‐1Met to overcome resistance to apoptosis in p53 mutated MM cells inducing ROS dependent cell death. Besides, both compounds showed antiproliferative activity in myeloma cells resistant to the Bcl2‐specific BH3‐mimetic venetoclax, inducing apoptosis in a Bak/Bax‐independent manner.

The in vivo anticancer activity of AF was assessed in a human RPMI8226 xenograft model by Sze et al.^42^ They demonstrated that AF was able to arrest tumor growth and induce apoptosis through TrxR inhibition.

Overall, these studies have strengthened the idea of a possible clinical use of AF. Indeed, very recently, AF, at a concentration ranging from 0.01 to 10 µM, was evaluated by Bonolo de Campos et al. in a “Direct Drug” screening with other 76 FDA‐approved oncology drugs and emerging therapeutics in 25 MM and 15 non‐Hodgkin's lymphoma cell lines and in 113 primary MM samples.^43^ The aim of the study was to correlate the drug sensitivities to clinical phenotype, genetic mutation, and transcriptional profiles to favor individualized therapeutic approaches in MM and to support clinical trials. AF turned out to have the broadest cytotoxicity in primary MM among with proteasome inhibitors, dinaciclib, selinexor, venetoclax, and histone deacetylating agents.

#### 2.1.2 Leukemia

In the last 40 years, several in vivo and in vitro studies have been published on AF as a potential anticancer agent for leukemia treatment. The first in vivo investigations were published in 1981 and 1985; these studies demonstrated the ability of this gold(I) compound to increase the life span of C57BL × DBA/2 F mice inoculated with the lymphocytic leukemia P388 cell line.^44^, ^45^ Later, the cytotoxic activity of AF was analyzed in acute promyelocytic leukemia (APL) model cells. This type of cancer is characterized by a block of differentiation and an accumulation of promyelocytic cells. All‐trans‐retinoic acid (ATRA) was utilized in the treatment of APL with a 90%–95% complete remission rate of patients.^46^ High doses of ATRA can restart cell differentiation even if 30% of patients relapse within 4–5 years.^47^ Several studies suggested a potential therapeutic benefit in treating APL with a combination of AF and a low dose of ATRA^48^ or 1,25(OH)2 vitamin D3^49^ (another differentiation‐inducing agent^50^ thus overcoming the harmful side effects caused by high doses of these drugs (i.e., ATRA resistance in relapsed APL patients and the hypercalcemic side effect and incomplete cell differentiation with 1,25(OH)2 vitamin D3^51^). In particular, Park et al. deciphered the molecular mechanism responsible for AF‐induced apoptosis demonstrating that AF (2 µM) proapoptotic effects in human HL‐60 cells depended on p38 mitogen‐activated protein kinase (MAPK) signaling activation.^52^ ROS induction by AF was proposed as a mechanism of p38 MAPK activation, since N‐acetyl‐l‐cysteine (NAC) inhibited p38 MAPK phosphorylation, preventing apoptosis.

Fiskus et al. attempted to provide rationale to AF repurposing for chronic lymphocytic leukemia (CLL) therapy.^53^ Standard therapy is based on myelotoxic chemoimmunotherapy.^54^ However, in case of relapse a different treatment is required as standard therapy is no longer effective. This type of cancer was suitable for AF repurposing because CLL cells have intrinsically higher levels of ROS.^55^ The authors demonstrated for the first time thatAF 1 µM triggered lethal oxidative stress through TrxR inhibition and ER stress response in cultured and patient derived primary CLL cells, including those with genetic features associated with poor clinical outcome. AF anticancer activity was also proved to be efficient in TCL‐1 transgenic mice, an in vivo model of CCL.^56^

Chen et al.^57^ evaluated AF on chronic myelogenous leukemia (CML) cell and animal models, as an alternative therapy to overcome acquired resistance to Imatinib mesylate (IM), a drug resistance mainly due to mutation in Bcr‐Abl.^58^, ^59^ They demonstrated that AF had antiproliferative activity in both Bcr‐Abl wild‐type and resistant Bcr‐Abl‐T315I CML cell lines as well as in mouse IM‐resistant xenograft models, thus illustrating AF ability to overcome drug resistance. AF‐induced apoptosis depended on both Bcr/Abl signaling downregulation and proteasome‐dependent caspase activation, while ROS were not involved in cell death process. In a subsequent study, Clapper et al. demonstrated that in imatinib resistant CML cells the TrxR/Trx system was upregulated.^60^ The inhibition of the TrxR enzyme by AF and the gold compound [Au(d2pype)2]Cl decreased Bcr‐Abl and proto‐oncogene c‐myc protein expression leading to apoptosis also in IM resistant CML cells. The obtained results highlighted the effectiveness of TrxR targeting in overcoming IM resistance as well as the crosstalk between the TrxR system and Bcr‐Abl signaling pathway.

Acute lymphoblastic leukemia (AAL) is the most frequent neoplasm in childhood^61^ and, as various other cancers, it is characterized by an unbalanced redox homeostasis.^62^ For this reason, Haß et al.^63^ selected different ROS inducers to sensitize ALL cells to therapeutic treatment based on the second mitochondria‐derived activator of caspases (Smac) mimetic LCL161 that antagonizes inhibitor of Apoptosis (IAP) proteins. They used AF alone or in combination with LCL161 to target TrxR, Erastin to inhibit the cystine/glutamate transporter XCT required for GSH production and buthionine sulfoximine (BSO) as specific inhibitor of γ‐glutamylcysteine ligase (γ‐GCL).^64^ The results pointed out the effectiveness of this approach in being able to prime ALL cells for LCL161‐induced cell death. The thioredoxin system was also evaluated as a novel therapeutic target against B‐cell acute lymphoblastic leukemia (BCP‐ALL) by Fidyt et al.^65^ This blood cancer is characterized by genetic heterogeneity and abnormal expansion of immature B cells. The currently available chemotherapy is effective in most cases. However, patients harboring certain genetic lesions, such as MLL rearrangements or Bcr‐Abl1 fusion, are resistant and need novel therapeutic approaches. The authors used AF and adenanthin (ADE), to inhibit the thioredoxin system. The results showed that these compounds triggered oxidative stress and ER stress leading to cell death, both in BCP‐ALL cell lines, pediatric and adult BCP‐ALL primary cells, including primary cells cocultured with bone marrow‐derived stem cells. AF efficacy was also demonstrated in vivo using a patient‐derived xenograft model. Recently Liu et al. identified a mitochondrial isoform of TrxR3 as a new target of AF.^66^ The authors found that TrxR3 was overexpressed in myelomonocytic leukemia MV4‐11 cells resistant to sorafenib (MV4‐11R) in comparison to parental cell line and furthermore, patients with high‐risk acute myeloid leukemia showed high expression of this enzyme and worse survival in comparison to low‐risk patients. They demonstrated, both in vitro and in vivo models, that TrxR3 high expression conferred sorafenib resistance and that AF was able to overcome resistance.

#### 2.1.3 Lymphoma

To our knowledge, the first study carried out on AF anticancer activity on lymphoma cell lines was published by Cox et al.^67^ They demonstrated that AF treatment activated apoptotic signaling through a Bcl‐2 homologous antagonist/(Bax/Bak)‐dependent mechanism associated to TrxR inhibition and peroxiredoxin3 (Prx3) oxidation in Jurkat T‐lymphoma and U937 monocytic cell lines. Celegato et al. investigated repurposing AF for refractory classical Hodgkin lymphoma (cHL).^68^ Its antitumour activity was demonstrated both in vitro and in vivo tumor models. AF (0.1–10 µM) has showed antiproliferative effects in L‐1236, L‐428, KM‐H2, HDLM‐2, and L‐540 cHL‐derived cell lines, in L‐540 gemcitabine‐resistant and HDLM‐2 brentuximab resistant cells. Furthermore, AF showed a synergistic activity with three chemotherapeutic drugs widely used in cHL treatment such as doxorubicin, cisplatin, and gemcitabine. This cytotoxicity was also demonstrated in L‐540 gemcitabine resistant–derived tumor xenograft. The proposed mechanism of action encompassed the inhibition of TrxR, ROS increase, and apoptotic pathway activation.

ROS‐induced apoptosis was also proposed as main mechanism in other studies where AF was used alone or in combination with other pro‐oxidant compound as l‐ascorbate, used at pharmacological concentration of 200 µM, and BSO.^69^, ^70^ Finally, Wang et al. repurposed AF to treat TP53‐mutated or (PTEN) deleted refractory B‐cell lymphoma.^71^ In several refractory mantle cell lymphoma (MCL) and diffuse large B‐cell lymphoma (DLBCL) models, they demonstrated that AF inhibited TrxR, increased ROS production and apoptosis. Its cytotoxicity was mainly effective in TP53‐mutated or PTEN‐deleted lymphomas strengthening the hypothesis of AF repurposing for treatment of TP53‐mutated or PTEN‐deleted refractory B‐cell lymphoma.

### 2.2 Breast cancer

Breast cancer is the most common female cancer and the second most common cause of cancer death in women. Up to 20% of breast cancers are “triple negative” tumors (TNBC) as the three most common types of receptors known to fuel most breast cancer growth—estrogen, progesterone, and the receptor tyrosine‐protein kinase (HER‐2/neu) gene—are not present in cancer cells. TNBC are not treatable with antihormone therapy, thus chemotherapy and radiation therapy represent the only possible form of treatment. Unfortunately, most patients develop resistance to these interventions and there is an unmet clinical need to find novel targeted and combination therapies.

Several studies have recently investigated the ability of AF to counteract breast cancer both in vitro and in vivo. In most of them, AF has been used in combination with at least one other drug to increase oxidative stress and induce cell death. Scarbrough et al. reported that simultaneous targeting of both GSH with BSO and TrxR with AF (0.5 µM), enhanced chemo‐sensitization to 17‐Allylamino‐17‐demethoxygeldanamycin (17AAG) (an experimental chemotherapeutic agent believed to increase oxygen free radicals) and 2‐deoxy‐d‐glucose (2DG) combined treatment in MDA‐MB 23 and SUM159 cell lines.^72^ The same research group also proved that inhibition of TrxR by AF (1 µM) potentiated cancer cells clonogenic killing induced by inhibiting both glycolysis (with 2‐DG) and pentose phosphate cycle (with dehydroepiandrosterone DHEA).^73^ Similar results were also obtained using PC‐3 prostate cancer cell lines^73^ Kim et al. demonstrated that AF (0.5–1 µM) was able to inhibit cell proliferation and cell growth on soft agar in MDA‐MB 23 through inhibition of STAT3 phosphorylation, in a ROS‐dependent fashion.^17^ Other two studies on triple negative cell lines indicated that 0.5 µM AF in combination with vitamin C (used as prooxidant) or with vitamin C plus menadione induced cell death mostly through the generation of oxidative stress, strengthening the unbalance of the redox homeostasis as main mechanism of AF action.^74^, ^75^ On the other hand, in 2014 Liu et al. reported for the first time that proteasome inhibition, and not ROS production, was required for AF‐induced cytotoxicity and apoptosis both in vitro and in vivo model.^76^ They demonstrated that AF inhibited the activity of deubiquitinases (DUBs) associated to 19S regulatory subunit of proteasome in MCF‐7 and in HepG2 (hepatoma cell line). This caused and accumulation of ubiquitinated proteins that was already detected after 3 h of 0.5 µM AF treatment indicating the proteasome inhibition occurred early and at low dose of the drug.

Very recently, AF was used in combination with an antiprogrammed death‐1 ligand (PD‐L1) antibodies to target this key immune checkpoint.^77^ In fact, the immune system plays a critical role in controlling neoplastic transformation and progression and targeting immune checkpoints constitutes a valid possibility to treat tumors refractory to conventional therapies. One of these checkpoints is represented by programmed death‐1 (PD‐1) receptor and PD‐L1. PD‐1 receptor is expressed on the surface of T‐cells and displays immunoinhibitory activity. Cancer cells express PD‐L1 on their surface and interact with cytotoxic T‐cells, inhibiting their function and evading host immunosurveillance. In several cancers, PD‐L1 expression increases in response to chemotherapy and leads to drug resistance; in triple negative breast cancer it is overexpressed in approximately 20%–30% of patients and is associated with poor prognosis.^78^ The authors demonstrated that AF induced cell death in different breast cancer cell lines (with IC_50_ values ranging between 0.5 and 2 µM in TNBC cells) and impaired the growth of TNBC‐derived spheroids. AF treatment exerted significant in vivo antitumour activity in multiple TNBC models including the syngenic 4T1.2 model, MDA‐MB‐231 xenograft and patient‐derived tumor xenograft. Interestingly the authors showed that AF alone failed to completely eliminate tumors in all in vivo models due to AF‐induced increased PD‐L1 expression through the ERK1/2‐myc axis. This observation led to the rationale of the implementation of a combined AF and anti‐PD‐L1 antibodies treatment. Combined treatment synergistically impaired the growth of 4T1.2 primary tumors, overcoming the AF‐induced increased PD‐L1 expression.

In addition, Rodman et al. have shown that AF is also capable of potentiating the response to radiation therapy both in vivo and in vitro.^79^ They demonstrated that combined inhibition of GSH synthesis with BSO and TrxR with AF‐enhanced cancer cell clonogenic killing and radiation responses via a mechanism that could be inhibited by N‐acetylcysteine (NAC), thus depending on thiol redox state. Furthermore, pre‐treating breast cancer stem cells with AF sensitized them to radiation. Finally, in vitro results were confirmed by in vivo experiments showing that in human breast cancer xenografts, combined administration of AF and BSO before radiation significantly increased survival rate and decreased the number of cancer stem cells. Very recently, Kim et al. found that the RNA binding protein NONO was bounded to STAT3 mRNA and to STAT3 protein, increasing its stability, transcriptional activity and contributing to STAT3 oncogenic function.^80^ Using a high‐throughput drug screening, they revealed that AF was a potential inhibitor of the expression of NONO, suggesting a new way in which AF could carry out its antitumour activity.

### 2.3 Colon cancer and gastric cancer

Colon cancer is one of main cancer types that lead to cancer‐related death worldwide.^81^ Chemotherapy is an important treatment approach for this pathology; however, patients have few treatment options after two cycles of therapy. Hence, the discovery of new treatments for colon cancer care is mandatory. AF (0.5 µM) was combined with 5Z‐7‐oxozeaenol (5 µM), a molecule able to inhibit the transforming growth factor β‐activated kinase 1 (TAK1), leading to colon carcinoma cell death both in vivo and in vitro.^82^ The inhibition of TrxR activity and the increased Trx oxidation were proposed to be responsible for the enhancement of sensitization of cancer cells to 5Z‐7‐oxozeaenol. Moreover, AF has been used in combination with the anti‐inflammatory drug celecoxib (CE) in different colon carcinoma cell lines.^83^ The findings show that AF/CE combination greatly enhanced AF therapeutic efficacy both in vitro and in vivo. Drug mixture generated oxidative stress that inhibited hexokinase, induced TrxR oxidation and modifies mitochondrial redox state. Particularly in mitochondria, AF/CE promoted the oxidation of TrxR2 and the degradation of the cytochrome c oxidase subunit II, a component of complex IV of the electron transport chain. As consequence, mitochondrial respiration and ATP production resulted inhibited.

5‐FU is widely used in chemotherapy for colon cancer, but drug resistance limits its clinical effectiveness. In a recent study, Liu et al. found that nuclear FOXO3 was decreased in 5‐FU‐resistant SW620 and HCT‐8 (SW620/5‐FU and HCT‐8/5‐FU) cells, and that overexpressing FOXO3 could significantly reverse 5‐FU resistance.^84^ FOXO3 is a transcription factor with key role in cell cycle, apoptosis, aging regulation, and tumor suppression. Several protein kinases (IkB kinase, Akt, MAP) phosphorylate FOXO3, promoting its translocation from the nucleus to the cytosol where it is degraded via the ubiquitin‐proteasome pathway.^85^ Its inhibition leads to tumor development and progression, suggesting that FOXO3 reactivation could be a promising strategy to develop anticancer therapeutic drugs. The authors demonstrated that TrxR1 was the key protein participating in the regulation of FOXO3‐mediated 5‐FU resistance. Thus, they used AF as FOXO3 agonist and TrxR1 inhibitor to overcome 5‐FU resistance both in vitro and in vivo models.

The possible beneficial effect of AF during radiation treatment has been analysed in subcutaneous CT26 colon tumors, in normal gastrointestinal epithelium of mice and in human organoids formed using malignant and nonmalignant tissues from the same patient.^86^ Indeed, one of the disadvantages in the use of radiotherapy in colon carcinoma therapy is the elevated radio sensitivity of normal intestinal epithelium. The results showed that AF pretreatment prevented radiation toxicity in normal cells in all these models. In fact, AF caused cell cycle arrest and p53/p21 pathway activation, leading to reduction of the irradiation‐induced DNA damages and increasing cell survival. On the other hand, in malignant cells AF inhibited proteasome activity and induced endoplasmic reticulum stress/unfolded protein response, activating apoptosis.

Concerning gastric cancer, Peng et al. demonstrated that in gastric cell lines, the natural compound piperlongumine (PL), known to induce ROS production, enhanced AF cytotoxicity.^87^ In particular, 4 µM AF induced ROS increase, ER stress and mitochondrial dysfunction leading to apoptosis. The combination of AF and PL resulted in an increase of cell death in vitro and in a reduction of tumor size in vivo.

### 2.4 Lung cancer

Lung cancer is the leading cause of cancer‐related deaths in men and women.^88^ In particular, non‐small cell lung cancer (NSCLC) accounts for about 85% of all lung cancers and has a poor prognosis with a 5‐year survival rate of 17%. Currently common therapies for NSCLC include platinum‐based chemotherapy with concurrent radiotherapy but cancer cell resistance limits treatment utility.

Fath et al. investigated AF efficacy in combination with 2DG and carboplatin in both in vitro and in vivo models.^89^ First, the authors demonstrated that 2DG enhanced carboplatin‐induced clonogenic cell killing and that this effect was almost abolished by NAC, indicating that 2DG plus carboplatin effect in lung cancer cells was mediated by disruptions of thiol metabolism. Second, they showed that inhibiting both GSH synthesis with BSO and TrxR activity with AF resulted in the clonogenic killing of A459 and H292 cell lines (with AF 5 and 0.5 µM, respectively), while single drug treatment had no effect on A459 cell line and low effect on H292. These results represented the rationale to treat cancer cells with a combination of AF + BSO and 2DG + carboplatin. As expected, this treatment caused a decrease of the clonogenic survival of 2DG + carboplatin treated A549 cells as well as increasing thiol oxidation that was inhibited by NAC. Most importantly, in A549 xenograft mice, the combination of AF + BSO + carboplatin resulted in a highly significant decrease in tumor growth rate when compared to control and to treatment with BSO + AF or carboplatin alone.

Fan et al. demonstrated that selenocysteine (SeC) acted as a natural inhibitor of TrxR1 in vitro and in vivo to enhance AF‐induced human lung cancer cells killing through ROS‐mediated apoptosis and inactivation of Mitogen‐Activated Protein Kinase 1 (Erk) and Akt pathways.^90^ Also, in this study, treatment of A459 with AF alone (6 µM) was not able to induce cell death, but pretreatment with SeC resulted in a severe cell death increase. The data were also confirmed in vivo: indeed, A459, xenograft growth in nude mice was more effectively inhibited by combined treatment with SeC and AF than with AF alone.

PI3K/Akt pathway plays a pivotal role in cancer cell growth and survival and inhibition of this pathway may represent a tool in cancer therapy. It has been reported that a large subset of NSCLC cells does not respond to Akt inhibitors,^91^ thus Dai et al. used a genome‐wide siRNA library screening to identify synthetic lethality loci with Akt inhibitor MK2206.^92^ Among these loci, they identified the TrxR1gene and demonstrated that inhibiting TrxR1 with siRNA or AF, sensitized lung cancer cell lines resistant to MK2206 to treatment with this drug. The combined treatment with AF 0.1 µM induced apoptosis by ROS activation of mitogen‐activated protein kinase 8 (JNK). Furthermore, in H1993 xenograft nude mice, treatment with MK2206 or AF as single drugs had no effect on tumor growth, but their combination significantly inhibited tumor growth and significantly prolonged animal survival. The same research group also investigated the cytotoxicity of AF alone in 10 different NSCLC cell lines and they found that AF sensitivity ranged from less than 1 µM to up to 2 µM.^93^ They demonstrated that sensitive cells presented lower expression of TrxR1 than resistant cell lines. The authors selected representative sensitive (Calu3 and HCC366) and resistant (A549) cell lines for further experiments. In the sensitive cell lines, a high‐throughput protein profiling analysis of 214 proteins and phosphoproteins was performed and several key nodes in the PI3K/Akt/mTOR pathway resulted repressed. Since the transient overexpression of the TrxR1 protein partially reversed this repression, they postulated that TrxR1 may participate in the regulation of PI3K/Akt/mTOR pathway and that high TrxR1 levels can attenuate AF‐induced inhibition of the PI3K/Akt/mTOR pathway. The activity of AF was confirmed in vivo in Calu3 xenograft nude mice. The same group also proved that the antitumour activity of a combination of TUSC2 gene (tumor suppressor candidate 2) delivered by nanovesicles and erlotinib (epidermal growth factor receptor [EGFR] inhibitor) was enhanced by AF both in vitro and in vivo.^94^

Hou et al. investigated the effect of AF on cancer stem cell side‐population (SP).^95^ SP represents a subpopulation of stem‐like cancer cells that have a pivotal role in drug resistance due to their high expression of the ATP‐binding cassette transporter ABCG2 involved in drug export. The authors found that A549 cell line, contained a considerable amount of SP and AF could effectively deplete SP cells by increasing ROS generation. Interestingly, they proved that AF inhibited the glycolytic enzyme hexokinase leading to ATP depletion and decreasing the activity of ABCG2 pump. Furthermore, the synergistic effect of AF and the chemotherapeutic agent adriamycin was proved both in vivo and in vitro.

Trx/TrxR and glutathione/glutathione‐disulfide reductase (GSH/GSR) are the most relevant cellular antioxidant systems and functional deficiency in one of these systems renders cells dependent on the other system for survival.^96^ Thus, it has been hypothesized that cells harboring a compromised GSH/GSR system could be more sensitive to TrxR inhibition. Yan et al. found that GSR gene is delete in 6% of lung adenocarcinoma. To investigate whether this deletion could sensitize cells to AF treatment, the authors evaluated AF sensitivity in several cell lines with different expression of genes involved in GSH homeostasis and in AF resistant A459 cell line in which GSR gene was knocked out or silenced. In all these conditions the authors demonstrated that the alteration of GSH homeostasis was correlated with increased sensitivity to AF treatment.^97^ Moreover, they also demonstrated in vivo activity of AF in two lung cancer patient‐derived xenograft models with relatively low levels of GSR and glutamate‐cysteine ligase catalytic subunit expression.

Activating EGF mutations represent a driven force in lung tumor progression and limit the therapy based on EGFR inhibitors. It is interesting to note that AF (0.25 µM) enhanced ibrutinib activity in EGFR mutant non‐small cell lung cancer cells by inhibiting the expression or phosphorylation of multiple key nodes in Akt/mTOR and MEK/ERK pathways.^98^ Ito et al. proved that AF in combination with IPA‐3, an inhibitor of p21 activate kinase (PAK1) which is in turn regulated by PKCiota, had a highly synergistic effect in EGFR or KRAS mutant adenocarcinoma and squamous cell carcinoma cell lines and decreased tumor volume in mice models.^99^ In these cell lines the combination of IPA‐3 and AF abrogated the expression and/or phosphorylation of serine/threonine‐protein kinase PAK1, PKCiota, ERK 1/2, mTOR, Akt, and transcriptional coactivator YAP1 proteins.

Liu et al. developed a high‐throughput drug screen strategy to identify new drugs that can synergistically enhance antitumour efficiency of cisplatin in chemoresistant small cell lung carcinoma (SCLC) cells.^100^ Among the FDA‐approved drug library of 1092 compounds they found that AF was able to overcome cisplatin resistance. In this study the authors demonstrated that AF synergized with cisplatin resulting in ROS overproduction, which in turn led to mitochondrial dysfunction and DNA damage in SCLC. The combination resulted in significant reduction of tumor growth also in vivo.

### 2.5 Ovarian cancer (OC)

OC is the most lethal gynecological disease and is characterized by heterogeneity, high risk of relapse and development of platinum resistance that result in poor prognosis. The cornerstone of treatment is maximal‐effort surgical cytoreduction combined with cytotoxic chemotherapy. An estimated 80%–85% of patients with OC who achieve full remission following first‐line platinum‐based therapy will develop recurrent disease and median survival for these patients ranges from 12 months to 24 months.^101^ Except for bevacizumab and poly ADP‐ribose polymerase inhibitors, approved for BRCA1/2 mutated patients and for platinum‐sensitive patients without BRCA 1/2 mutation, there are few other options for women with platinum‐resistant OC.

The first articles that report a cytotoxic activity of AF on ovarian cancer cells were published by Marzano^102^ and Rigobello.^103^ In these studies, the authors demonstrated that AF could overcome cisplatin resistance in the cisplatin‐resistant variant (C13*) of the sensitive 2008 cell line. AF, by inhibiting TrxR activity, induced ROS mediated activation of apoptosis. The authors also explored the effect of AF (1 µM) in combination with selenite that is a powerful pro‐oxidant compound at moderate to high concentrations. Selenite pretreatment induced a dramatic increase in AF cytotoxicity in both resistant and sensitive cells. Cytotoxic AF properties have been investigated by 2D‐gel proteomic approach.^104^, ^105^ These studies, performed on A2780 sensitive and cisplatin‐ resistant (A2780R) cells showed that AF (0.5 µM) affected the proteins involved in redox balance (peroxiredoxin 1 and 6, thioredoxin‐like 1 protein) and in protein degradation.

In 2013, Wang and collaborators found a new AF target: the PKCiota protein a member of the protein kinase C family.^106^ The authors cultured ES2 and SKOV3 ovarian cell lines in stem cell medium to obtain cells with tumorigenic tumor initiating cell (TIC) properties (TICs cells). TIC properties include expression of stem‐related genes, clonal expansion, enhanced transformed growth, resistance to chemotherapy. These characteristics resemble properties of ovarian cancer stem cells that play a key role in relapse following systemic chemotherapy.^107^ These authors demonstrated that ovarian TICs require PKCiota for clonal expansion, enhanced anchorage‐independent‐growth, and tumor initiation in vivo. Pharmacologic inhibition of PKCiota with AF blocks the TIC phenotype in vitro and TIC‐mediated tumor formation in vivo. PKCiota inhibition was achieved with AF 1 µM treatment for 24 h. This inhibition was probably direct, since it has been previously demonstrated that two analogues of AF, namely aurothiomalate and aurothioglucose, interact with a critical cysteine residue within the PKCiota PB1 (Phox and Bem 1) domain leading to inhibition of oncogenic PKCiota signaling.^108^

Two other interesting articles focused on the inhibition of FOXO3 pathway. Park et al. showed that AF at 0.1 µM for 48 h downregulated the expression of IkB‐kinase, one of the kinase responsible of FOXO3 phosphorylation^109^ and induced the nuclear translocation of FOXO3 in SKOV3 cells that are p53 null.^22^ Furthermore, FOXO3 silencing caused a decrease in apoptosis indicating that in this cell line, AF induced apoptosis through FOXO3. The same research group reported that the anticancer activity of AF via FOXO3 regulation could be enhanced by inhibition of the expression of mucin 4 (MUC4).^110^ MUC4 is a membrane glycoprotein, it is overexpressed in several cancers and promotes tumor formation, tumor aggressiveness, and poor outcomes in various types of epithelial carcinomas, including ovarian cancer.^111^ AF combined with MUC4 silencing decreased the expression of receptor tyrosine‐protein kinase erbB‐2 (Her2) and Akt phosphorylation, leading to inhibition of the Her2/Akt/FOXO3 pathway and thus inhibiting FOXO3 translocation from nucleus to cytosol.

It is well known that BRCA 1/2 mutation predisposes women to increased risks of developing ovarian and breast cancers. Oommen et al. demonstrated that in OVCAR5 and SKOV3 cells, BRCA1 silencing increased AF cytotoxicity and that AF‐induced DNA double strand brakes in a ROS‐dependent manner, thus demonstrating that BRCA1 deficiency renders tumor cells more sensitive to AF.^112^ Hyter et al. tested the cytotoxicity of AF alone or in combination with HSP90 inhibitors in ten ovarian cancer cell lines founding that there were remarkable differences in AF sensitivity in different lines.^113^ They utilized A1847 and PEO4 as representative cell lines for the sensitive and resistant groups respectively and they demonstrated a synergic effect of AF and the HSP90 inhibitor AUY922. They used the most effective synergic concentration to evaluate apoptosis, protein ubiquitination and expression level of Nrf2, a transcription factor that binds to the antioxidant response element of several detoxifying genes. They also created multigene signatures linked to sensitivity or resistance and sought out the prevalence of these genes in publicly available TCGA databases. This approach pointed out that the resistant cell lines were more like clinical samples and were more appropriate models. Recently Marzo et al. analysed the in vitro and in vivo properties of AF and an AF analogue, the iodido(triethylphosphine)gold(I) complex (Et_3_PAuI) in which the thiosugar ligand was simply replaced by one iodide ligand.^114^ The two compounds exerted similar cytotoxic and proapoptotic effect on A2780 human ovarian cancer cells in vitro. However, when evaluated in a preclinical orthotopic model of ovarian cancer, Et_3_PAuI produced a far superior anticancer action than AF, inducing almost complete tumor remission. This result confirmed that the thiosugar ligand is not mechanistically indispensable and that the [Et3PAu]^+^ moiety is likely to be the true pharmacophore.

### 2.6 Other cancers

The literature contains several other studies concerning the use of AF as a cytotoxic and potentially antitumor agent in other cancer types. Taken together they confirm the possible broad spectrum of application of this drug.

Concerning hepatoma, Kim et al. reported that 2 μM AF was able to inhibit IL‐6 activation of JAK1‐STAT3 pathway in a ROS‐independent fashion.^16^ Huang et al. found that AF (0.2 μM) in combination with Disulfiram (an aldehyde dehydrogenase, in clinical use to treat alcoholism) synergistically induced proteasome‐associated DUBs inhibition, reticulum endoplasmic (RE) stress and apoptosis in HepG2 and SMMC‐7721 cell lines.^115^ They also proved that this mechanism was ROS‐independent since scavenging of ROS by 100 µM Vitamin C failed to block cell death. As already shown for other cancer types, also in hepatocellular carcinoma TrxR1 expression positively correlated with advanced clinical staging and poorer patient survival.^116^ Thus, inhibition of TrxR1 represents a valid tool to counteract cancer progression. Lee et al. demonstrated that AF treatment sensitized several hepatoma cells lines toward conventional therapeutic Sorafenib and furthermore, AF alone was able to inhibit tumor growth in vivo.^117^ Hwang‐Bo et al. demonstrated that 1 µM AF was able to induce oxidative stress in Hep3B cells and that the natural compounds morin and sulforaphane enhanced its anticancer activity.^118^, ^119^ They showed that cotreatment with morin induced activation of both extrinsic and intrinsic pathways of apoptosis by upregulating death receptors DR4 and DR5 and by modulating Bcl2 family proteins. Furthermore, AF plus sulforaphane displayed a synergic induction of ROS‐mediated PI3K/Akt signaling.

Annexin A5 (ANXA5) has a pivotal role in mediating cytotoxicity, apoptosis, and anti‐inflammatory effects.^120^ Park et al. suggested that, in PC3 prostate cancer cell lines, AF (2.5 µM) induced ANXA5 expression and translocation into mitochondria where it possible participated in VDAC oligomerization and activation of caspase‐3.^121^ More recently, the same group demonstrated that AF suppressed cyclooxygenase 2 (COX‐2) expression through ANXA5 induction, thus inhibiting COX‐2 mediated inflammation.^122^ In PC‐3 cells, induction of ANXA5 by AF was also proposed to enhance apoptosis by suppressing the expression of plasminogen activator inhibitor (PAI‐2),^123^ a protein involved in tumor vascularization, cell migration, and tumor metastasis.^124^

Androgen receptor is often overexpressed in prostate cancer and is strongly linked to tumor growth and progression, thus, its targeting with competitive inhibitors or its down regulation is crucial for therapy. In this context, Liu et al. proved that AF (1 µM) promoted androgen receptor protein degradation and inhibited androgen receptor transcription in two androgen receptor‐positive cell lines, LNcap and 22RV1.^125^ Xu et al. demonstrated that the combination of enzalutamide (ENZ) and docetaxel (DTX) effectively inhibited the growth of prostate cancer cells that are resistant to either single drug.^126^ Thus, the authors generated a cell line resistant to both DTX and ENZ and demonstrated that AF could overcome resistance by inhibiting the growth of these drug‐resistant prostate cancer cells both in vitro and in vivo.

In prostate cancer as well as in other cancers, some regions of tumor microenvironments are often nutrient‐deprived and hypoxic because of aberrant cell proliferation and insufficient vascularization. Recently, Onodera et al. found that inhibitors of the redox system displayed preferential cytotoxicity to cancer cells in nutrient‐deprived conditions.^127^ AF treatment in PANC‐1 cells showed preferential cytotoxicity under nutrient‐deprived compared with nutrient‐sufficient condition with an EC50 of 0.64 and 2.18, respectively.

In literature there are only two studies concerning AF in treatment of head and neck squamous cell carcinoma (HNSCC).^128^, ^129^ Both were based on the prooxidative activity of AF. Overall, they demonstrated that inhibition of the Nrf2 pathway in addition to inhibition of γ ‐GCL and TrxR could effectively eliminate resistant cells.

The high‐throughput screen of FDA approved drugs was used to discover new therapies for osteosarcoma and Ewing sarcoma.^130^, ^131^ In both cases AF emerged as a possible alternative to traditional treatments both as a single drug and in combination with rapamycin or vorinostat (for osteosarcoma) and with ganetespib, an HSP90 inhibitor (for Ewing sarcoma).

Few papers dealing with AF and melanoma emerged from Pubmed research. To the best of our knowledge, AF cytotoxic effects were examined in a melanoma cell line (B16 melanoma cells) for the first time by Mirabelli et al. in 1985.^45^ Subsequently, Sachweh and colleagues proposed the inhibition of TrxR1 through compounds such as AF, as a possible therapy for malignant melanoma.^132^ This type of cancer is the most dangerous type of skin cancer due to drug resistance and relapse following treatment and, in most cases, it is characterized by the inactivation of the tumor suppressor p53 through dysregulation of upstream pathways. In the attempt to find a new melanoma therapeutic strategy, the authors screened for compounds that can reactivate p53 in melanoma cells. They identify the nongenotoxic compound MJ25 (2‐[2‐(1,3‐benzothiazol‐2‐ ylsulfonyl)ethyl]thio}−1,3‐benzoxazole) and proved that MJ25 could inhibit TrxR1. Thus they demonstrated that AF displayed effects comparable with MJ25 on several melanoma cells lines, confirming TrxR inhibition as a potential therapeutic strategy.

The AF efficacy has also been investigated in melanogenesis; Goenka et al. found that nontoxic concentrations of AF (0.25–1 µM) showed antimelanogenic activity in a dose‐dependent manner both in mouse B16F10 and MNT‐1 human melanoma cells lines, by inhibiting intracellular tyrosinase activity, decreasing cAMP levels and increasing immature melanosomes.^133^

Lastly, AF‐induced cell death via oxidative stress also in HeLa cervical cancer cells and was used in combination with 2‐DG and BSO (inhibitors of glycolysis and GSH synthesis, respectively) to treat highly glycolytic cervical cancer cell lines that were resistant to standard therapy with cisplatin plus pelvic irradiation.^134^, ^135^

## 3 DOSE‐DEPENDENT EFFECTS OF AURANOFIN THROUGH A SYSTEM BIOLOGY‐LIKE APPROACH

In the first part of this review, we reported an update on AF effects according to clinical applications in different cancers. Reporting the results obtained from different cell models, with different concentrations and exposition times, generates a fragmented vision of the AF mechanism of action. Thus, in this section we have summarized the major effects of AF in cancer cells by revisiting a large data set of transcriptomic data obtained with a wide range of cell models exposed to AF. The data are deposited in the connectivity maps (CMAP) website (https://clue.io/cmap)^136^ that, using transcriptional expression data (mRNA transcript abundance of 978 “landmark” genes), allows to probe the relationships between drugs and many cellular models of diseases. In practice, changes in gene expression in response to a genetic perturbation (knockdown or overexpression of a gene) or treatments with small molecules (perturbagenes) are compared by similarity to all perturbation signatures of the database (more than 1 million of available profiles) obtaining a “tau score” which allows to predict the effects of a perturbagene. CMAP is equipped with all the bioinformatic tools and transcriptomic data sets to compare the effects of different drug dosages and exposition times in many cancer cell lines.

**Figure 2:**
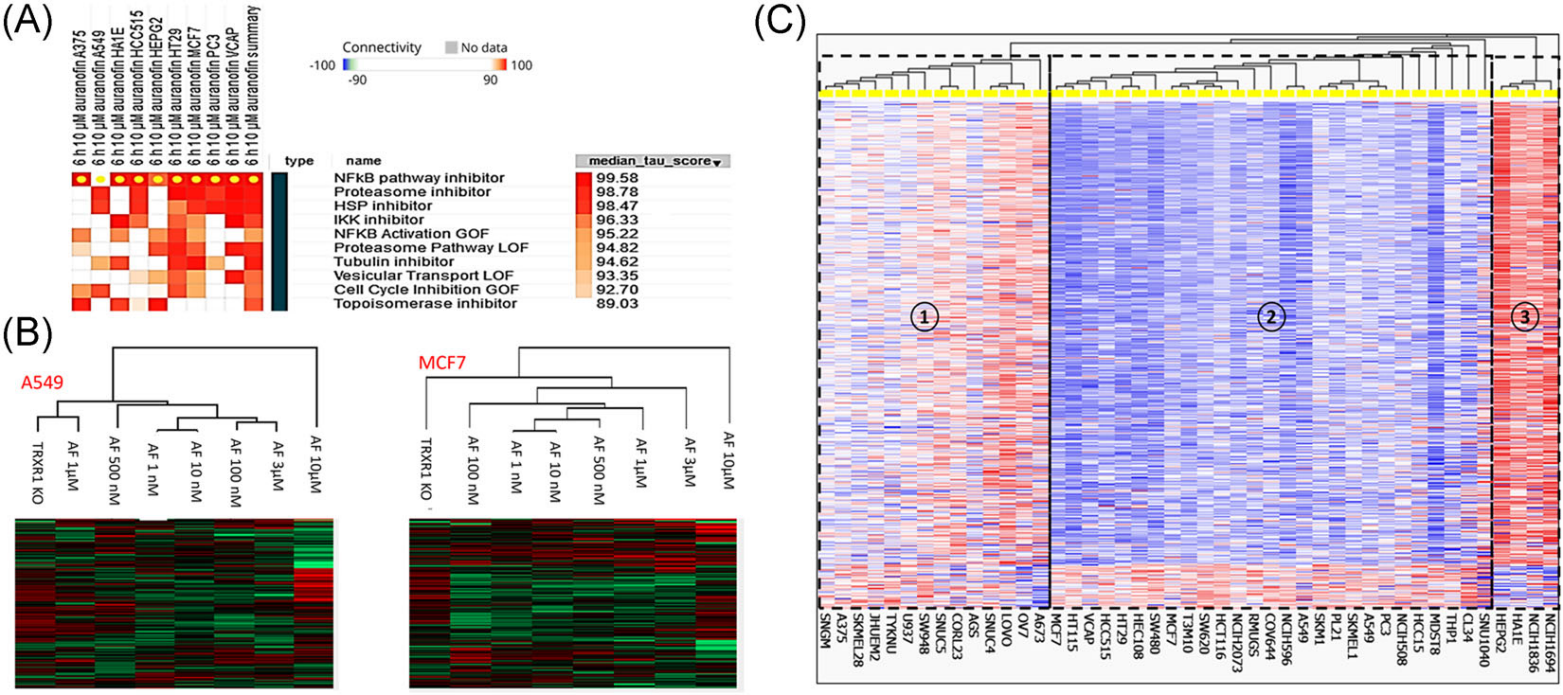
(A) Output of the connectivity map of AF realized with the transcriptomic data of nine cancer cell lines exposed to 10 µM of AF for 6 h. The median tau score is calculated from the tau score of each cell line. Only the “perturbagene” classes with tau score greater than 90 were reported. (B) Hierarchical clustering of the transcription profiles of A549 and MCF7 cell lines exposed to different AF concentrations together with the transcription profile of TrxR1‐silenced cells. (C) Hierarchical clustering of the transcription profiles of 45 cancer cell lines exposed to 10 µM of AF for 6 h. AF, Auranofin [Color figure can be viewed at wileyonlinelibrary.com]

Searching for the connectivities of AF with other “perturbagenes,” CMAP performs its prediction using the gene expression profiles of nine cell lines (A375, A549, HCC515, HEPG2, HT29, MCF7, PC3, HA1E, and VCAP) exposed for 6 h at 10 µM of AF which is generally considered a high dose. The highest AF connection is with the NF‐κB inhibition (Figure 2A). The NF‐κB inhibition effects of AF have long been known. Jeon et al. were the first to show that NF‐κB was inhibited with 5–10 µM of AF treatment for 4 h in lipopolysaccharide stimulated macrophages blocking IκB kinase (IKK) activity.^19^, ^27^ Later, Nakaya et al. reported that 0.05 µM of AF inhibited NF‐κB DNA binding and decreased NF‐κB nuclear protein level in U266 multiple myeloma cells.^18^ Moreover, Saei et al. recently proposed a proteomic based strategy to find candidate protein targets of AF, at 3 µM drug dose concentration, in HCT116 colon cancer cells.^137^ Also in this case, NF‐κB resulted to be the most probable gold target while TrxR1 was only in third position.

The second AF correlation predicted by CMAP is with proteasome inhibitor family. Inhibition of DUBs by AF (0.5 µM for 3 h) was firstly proposed by Liu et al. in HEPG2 and MCF7 cells.^76^ Later the same research group confirmed this finding in vitro and in vivo models of prostate cancer.^115^

On the other hand, in a recent proteomic study Zhang et al. compared the proteome profiles of HCT116 colon cancer cells treated with AF, with the proteasome inhibitor bortezomib and with the 19S DUBs inhibitor b‐AP15.^138^ They showed that AF dependent DUBs and proteasome inhibition only occurred at high dose of AF (>2.5 µM). Furthermore, AF (used at 1 and 5 µM concentration) induced a proteomic expression profile distinct from that of bortezomib and of AP15, both of which induced similar responses. Thus, AF‐dependent proteasome inhibition was considered an off‐target effect occurring at high dose of AF. Indeed, in the same study, monitoring the level of 1 HO‐1, considered a readout of Nrf2 activation, they observed that oxidative stress induced by TrxR1 inhibition occurred at lower concentration of AF (0.25 µM) compared to HCT116 cells IC50 (1.5 µM) and for this reason it should be considered the dominant mechanism underlying AF cytotoxicity. In our opinion, to try to interpret this set of sometimes conflicting results it should be considered that the cell type specific genetic background could also play a key role in determining the primary AF targets. This is confirmed by the comparison of the transcript expression profiles between different AF concentrations in two cancer cell lines and the effects of TrxR1 silencing, available in the CMAP data set. For example, A549 cells (lung cancer) exposed to 1 µM AF generate effects very similar to TrxR silencing whereas, MCF7 cells (breast cancer) exposed to different concentration of AF (1 nM to 10 µM) show low correlations with TrxR1 silencing (Figure 2B). Furthermore, considering the hierarchical clustering of the CMAP transcriptomic profiles of 45 cancer cell lines exposed to 10 µM AF for 6 h, three major clusters can be distinguished (Figure 2C). Cluster 1 is characterized by 14 cell lines showing very heterogeneous response to the treatment. Cluster 2 consists of 27 cell lines showing predominant transcript downregulation mostly linked to the cell cycle. Cluster 3 consists of four cell lines showing predominant transcript upregulation belonging to proliferative pathways such as PI3K–Akt and Jak–STAT3. Overall, these data confirm both dose and cell type‐dependent effects of AF and can provide a key to the interpretation of the different results reported in the studies summarized in previous paragraphs.

## 4 CONCLUSION AND PERSPECTIVES

Based on the studies performed on different cancer models, several mechanisms have been proposed to explain how AF alone or in combination with other drugs can exert its anticancer activity. These mechanisms can be partially overlapping and tumor and/or AF dosage dependent. As summarized in Table 1, AF EC50 after 24–48 h ranges between 0.5 and 2 µM in most of the cells. Most of the experiments were performed using AF in this range concentration for 12–24 h h. In some studies, AF was used at higher concentration (6–10 µM) for a shorter amount of time (2–6 h). These different approaches could lead to different pathway activations and complicate overall data interpretation as discussed in the previous paragraph. Since in humans, the plasma steady‐state physiological range of AF is 1–1.5 µM,^3^, ^4^ it is reasonable to hypothesize that in vivo, targets inhibited by low concentrations of AF have greater relevance.

**Figure 3:**
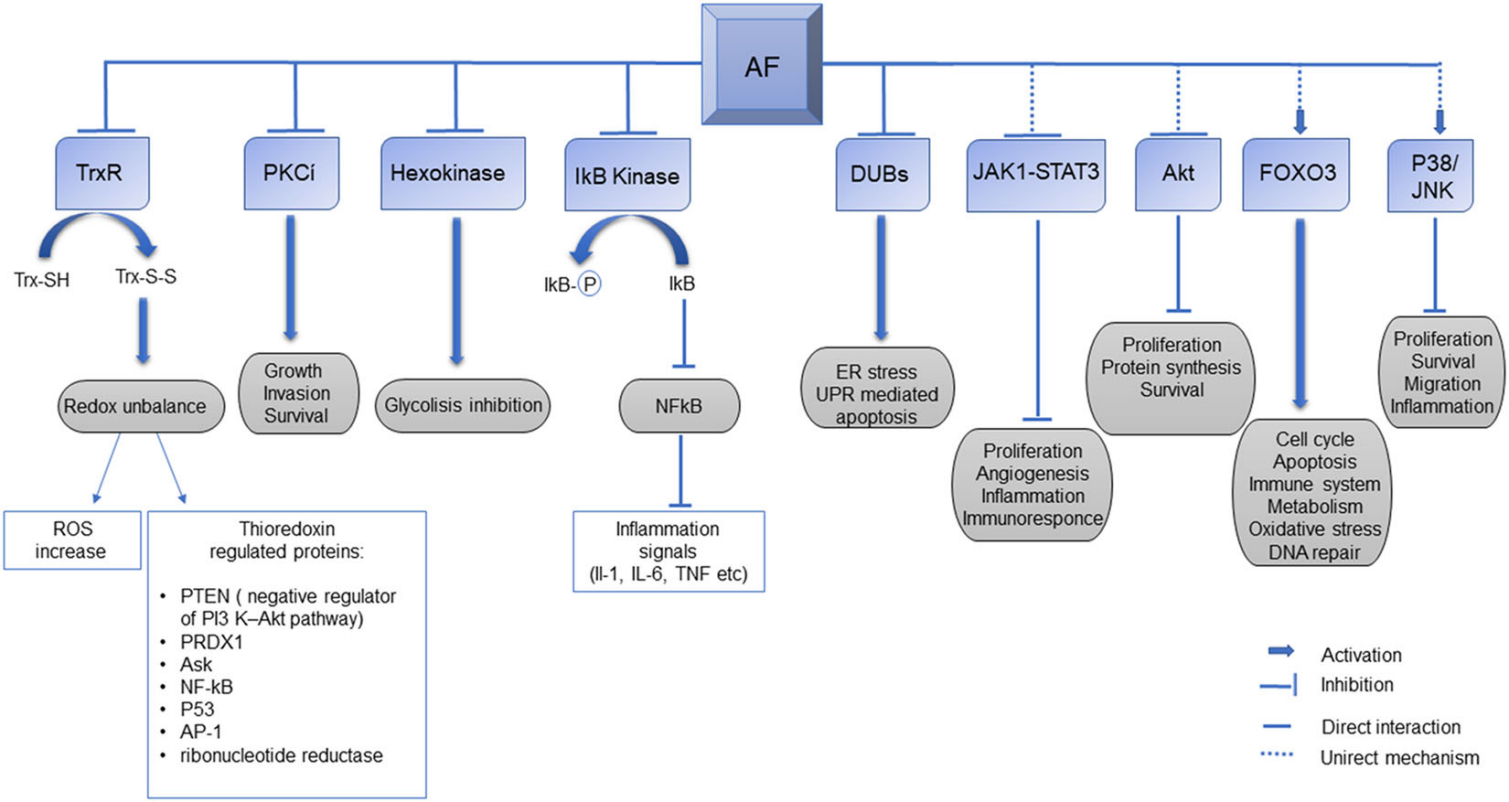
Overview of the main targets and the main signaling pathways affected by auranofin [Color figure can be viewed at wileyonlinelibrary.com]

In conclusion the main direct and indirect targets of AF are shown in Figure 3 and, in particularly, a direct interaction has been shown for the following proteins:

- TrxR inhibition
  The direct inhibition of TrxR was demonstrated for the first time in 1998 and up to date it represents the most proposed mechanism.^6^ In most articles, the inhibition of TrxR1 and the consequent increase of ROS has been proposed as the primary way by which AF induces cytotoxicity. In this context, it should be also mentioned that TrxR1 inhibition can influence the redox state of several proteins controlled by the TrxR/Trx system and therefore the ultimate effector of AF action may result difficult to identify.^13^, ^27^, ^57^ Another point to be considered is that not all cancers have the same expression level of this enzyme and thus, AF sensitivity may depend on this feature. In fact, several studies have shown that cell lines where TrxR1 expression levels were higher were also more resistant to AF cytotoxic activity.^31^
- IκB kinase
  IκB kinase was proposed as direct AF target by Jeon et al. in LPS‐stimulated macrophages. The same authors also demonstrated that AF binds Cys179 of IκB kinase, thus blocking its activity.^19^, ^27^ IκB kinase activity is strictly related to activation of NF‐κB. Indeed, IκB kinase phosphorylates IκB protein that binds NF‐κB and sequestrates it in the cytosol in an inactive state. Once phosphorylated, IκB dissociates from NF‐κB, that migrates in the nucleus where it carries out its transcriptional activity.^139^ In this review we have described several studies pointing out a reduction of NF‐κB activity upon AF treatment. This can impact on cancer progression, since NF‐κB regulates the expression of a plethora of genes involved in inflammation, oxidative stress and cell survival. In particular, since the correlation between tumor aggressiveness and inflammation has been extensively proved, the anti‐inflammatory properties of AF could be relevant if used in combination with other antitumour drugs.^140^ Interestingly, IκB is not the only substrate of IκB kinase, indeed Hu et al. found that it also phosphorylates the tumor suppressor FOXO3 that once phosphorylated, translocates to the cytosol and becomes inactive.^109^ Thus, IκB kinase inhibition by AF can evoke various responses whose importance for the cytotoxicity of the drug could depend on the specific cell type and, *in vivo,* on the tumor microenvironment.
- PKCiota
  PKCiota was indicated as AF target in 2013 by Wang et al.^20^ A possible direct interaction has been proposed since two analogues of AF, namely aurothiomalate and aurothioglucose, inhibit the enzyme, by binding a critical cysteine residue.^108^ Amplification of the PKCiota gene and elevated PKCiota mRNA level are observed in several cancers, that is, human primary lung squamous cell carcinomas, serous epithelial ovarian cancer, and cervical cancer.^141^ Furthermore, high PKCiota expression level is associated with decreased survival in patients with NSCLC, ovarian, bile duct, and prostate tumors, suggesting its possible role as prognostic marker.^142^ In consideration of all these aspects the AF inhibitory role on this kinase needs further investigation especially in cancer with high expression of this enzyme.
- Hexokinase
  The study of Hou et al. demonstrated that the purified enzyme hexokinase was inhibited by AF at 4–6 μM and that in vivo cellular glucose uptake, lactate and ATP production were also significantly suppressed by the same dose.^95^ Most cancer cells express high levels of hexokinase and this is implicated in the accelerated glycolytic flux.^143^ Furthermore, hexokinase (in particular the isoform II) can bind to mitochondria, where it plays additional functions such as cell death inhibition and autophagy regulation.^144^ Thus, targeting hexokinase is considered a promising antitumour strategy, but further evidence is needed to understand the possible involvement of AF especially if used at more physiological doses.
- Proteasome‐associated DUBs
  In 2014 Liu et al. demonstrated that the proteasome associated deubiquitinases UCHL5 and USP14 of the 16S regulatory subunit are inhibited by AF both in vitro and in vivo and that this inhibition played a key role in AF cytotoxicity.^76^, ^125^ As discussed in previous paragraph, Zhang et al. considered DUBs an off‐target effect occurring at high dose of AF.^138^ Further studies are needed to better understand dose‐dependent and/or cell type dependent inhibition of these targets.
  All the other proteins summarized in Figure 3 resulted affected by AF treatment but, as fa as we know, no evidence was provided regarding a direct interaction. It is reasonable to assume that their modulation is related to the inhibition of the direct targets previously described, but in several cases the signaling cascade has not been clarified. Furthermore, more than one mechanism can contribute to the ultimate effect of the drug.
  Overall, the studies described in this review support the use of AF in combination with the drugs normally used in the therapy of specific cancer, as a valuable aid to overcome resistance to chemotherapy and/or enhance the effectiveness of conventional therapies. Further efforts are needed to understand the effective mechanism through which AF performs its action on different types of cancer in relation to specific genome background and protein pattern expression. The studies reported in this review confirm the growing interest in AF drug repurposing and provide the basis for further insights that can support the translation of research in this field into clinical trials.

## Data Availability

The data that support the findings of this study are available in PubMed.
